# The Impact of Cooking on Antioxidant and Enzyme Activities in Ruichang Yam Polyphenols

**DOI:** 10.3390/foods14010014

**Published:** 2024-12-25

**Authors:** Haoping Liu, Hua Zhang, Mengting Geng, Dingxin Shi, Dongsheng Liu, Yanxiao Jiao, Zhiqiang Lei, You Peng

**Affiliations:** 1 College of Food science and Engineering, Jiangxi Agricultural University, Nanchang 330045, China; jingye20231109@126.com; 2 Jiangxi Ecological Chemical Engineering Technology Research Center, Jiujiang University, Jiujiang 332005, China; g2691401962@163.com (M.G.); sdx15554573852@126.com (D.S.); liudong2016@163.com (D.L.); jiaochem@126.com (Y.J.); 3 Department of Food Nutrition and Safety, College of Pharmacy, Jiangxi University of Chinese Medicine, Nanchang 330004, China; 20191002@jxutcm.edu.cn

**Keywords:** Ruichang yam polyphenols, antioxidant activity, α-Glucosidase, pancreatic lipase

## Abstract

In this study, the total polyphenol content (TPC), total flavonoid content (TFC), and biological activity of yam polyphenols (including free phenolics, conjugated phenolics, and bound phenolics) were investigated during home cooking. Polyphenol components were preliminary detected in raw yam by HPLC, including 2, 4-dihydroxybenzoic acid, syringic acid, vanillic acid, 4-coumaric acid, and sinapic acid. TPC and TFC of soluble conjugated polyphenols were the main phenolic compounds in Ruichang yam. Compared with uncooked yam, cooking times of 80 min and 40 min increased the TPC and TFC of multiple types of polyphenols, while cooking reduced the TPC and TFC of AHP (acid-hydrolyzed soluble conjugated polyphenols). All yam polyphenols exhibited good α-Glucosidase inhibitory activity; α-Glucosidase inhibitory activity was significantly higher for a cooking time of 120 min. Only some types of polyphenols had lower pancreatic lipase half-inhibition concentrations than orlistat when cooked. The pancreatic lipase of FPs (free polyphenols), BHPs (alkali-hydrolyzed soluble conjugated polyphenols), and ABPs (acid-hydrolyzed insoluble bound polyphenols) was the stronges when cooking for 80 min, and the pancreatic lipase inhibitory activity of AHPs and BBPs (alkali-hydrolyzed insoluble bound polyphenols) was strongest when cooking for 40 min. Pearson’s correlation coefficient analysis revealed that the TPC was positively correlated with the TFC, the IC_50_ value of α-Glucosidase was negatively correlated with the IC_50_ value of pancreatic lipase, and redox activity was positively correlated with the TPC and TFC, respectively.

## 1. Introduction

Chinese yams (Dioscorea opposite Thunb.) are a species of Dioscorea plants that produce edible tubers and are considered homologous plants of medicine and food [1]. Yam tubers contain diosgenin, polyphenols, proteins, and other chemical components, which have biological activities, including inhibiting obesity, regulating inflammation, anti-oxidation, and anti-diabetes [2,3]. Yams are widely cultivated in most areas of China. Commonly cultivated varieties of yams include the Jiangxi Ruichang yam and Henan iron rod yam [4]. Ruichang yam is one of the characteristic agricultural products in Ruichang, Jiangxi Province, with a planting history of 500 years [5]. Its tuber is of the stick type, with brown and yellow skin, and the upper part of the tuber is dense with fibrous roots [6]. The yam is mainly planted in Nanyang, Gaofeng, Guilin, Henglishan, and other townships in Ruichang [6,7]. The altitude of these areas is between 60 m and 600 m [8]. The soil is brown lime soil and red lime soil, and its pH is 6.4–7.0. Additionally, the soil is rich in organic matter and potassium contents [7]. Ruichang yam tuber, known as “Jiangnan ginseng” elegant, is rich in proteins, polysaccharides, starch, fat, vitamins, allantoin, and other components [4,6,9,10]. Ruichang yam exhibits biological activities that help regulate blood sugar and blood pressure, lower blood lipids, and enhance digestion and absorption. It also strengthens the spleen and stomach, alleviating lung issues and coughs, supporting kidney function, and promoting longevity [11]. In addition, Ruichang yam is usually used as a daily dish because of its unique nutritional value. It can be steamed, boiled, sauteed, simmered, deep-fried, and braised. It can also be cooked and eaten with some meat. As a local characteristic agricultural product, Ruichang yam is consumed by humans and is used as a raw material to process many products, such as yam tablets, yam powder, yam noodles, yam oil noodles, and yam wine.

Yams are usually prepared in various ways before consumption, and cooking significantly alters the chemical composition [12], which affects the concentration and biological activity of the compound. Different processing methods, such as cooking [13,14,15,16], baking, squeezing, and boiling [17], affect the phenolic components of foods and their potential antioxidant activity. Studies have shown that normal pressure boiling rather than frying resulted in the highest amount of bioaccessible phenolic compounds in yam [18].

Phenolic substances, according to their form of existence, can be classified as soluble free phenolics, soluble conjugated phenolics, and insoluble bound phenolics. Soluble conjugated phenolics are usually covalently combined with different metabolites, such as fatty acids (soluble esters) [19]. The covalent bonds between conjugated phenolic substances can be broken through acid and alkali hydrolyses [20,21]. The traditional solvent extraction method can only be used to extract soluble polyphenols, leaving a significant portion of polyphenols in the residue after extraction. Insoluble bound phenolics are polymerized and formed from high molecular weight (such as proteins, cellulose, hemicellulose, pectin, and the lignin of plant cell walls) or single, small molecular weight phenolics crosslinked with macromolecules [22]. In the study of bound phenolic compounds, alkaline hydrolysis and acid hydrolysis are the most commonly used methods for extracting insoluble bound phenolic polyphenols [17,22,23,24].

Some studies have shown that natural polyphenols can inhibit the activity of α-Glucosidase and pancreatic lipase [25,26,27]. α-Glucosidase is a carbohydrate hydrolase that can convert oligosaccharides and disaccharides into glucose [28]. α-Glucosidase can reduce the digestion and absorption of intestinal glucose, thereby supporting human health. Hence, α-Glucosidase is an important enzyme that plays a crucial role in preventing and managing diabetes [29]. Pancreatic lipase hydrolyzes dietary fats (such as triglycerides, cholesterol esters, and phospholipids) into lipolysis products (monoglycerides, cholesterol, and free fatty acids) [30], promoting the absorption of lipids by cells. Orlistat is a drug used to treat obesity, mainly by inhibiting the activity of pancreatic lipase [31]. Therefore, pancreatic lipase inhibition is an investigational mechanism for identifying potential anti-obesity drugs.

At present, many studies have shown that Chinese yams have certain edible and medicinal values. However, the effect of the long-term consumption of Chinese yams on some biological activities, such as antioxidant activity, hypoglycemic activity, and lipid-lowering activity of Ruichang yam, has not been reported. Therefore, we extracted free, conjugated, and insoluble bound phenolic compounds from Ruichan yam using a boiling method and evaluated the effects of boiling time on the content, antioxidant activity, α-Glucosidase, and pancreatic lipase activities of Ruichang yam.

## 2. Materials and Methods

### 2.1. Preparation of Cooked Chinese Yam

The cultivation conditions of Chinese Ruichang yam are very strict, requiring the use of local land that has not been cultivated in five years that is planted in the red soil of the mountain slopes. From sowing to harvesting, rapeseed oil meal is used to fertilize Ruichang yam to increase nutrients. Ruichang yam is sown at the beginning of winter every year, and harvest begins around October of the next year, which makes it an annual crop. The climate in the growing area is subtropical monsoon, with high temperatures and rain in summer and low temperatures and little rain in winter, so extra watering is needed when the weather is dry. The yam selected in this experiment was the Ruichang yam, which is harvested under the aforementioned cultivation conditions and purchased from Ruichang Jiangxi Province. The peeled yams were ground using a wall breaker (Midea, Hangzhou, China) and mixed with an appropriate amount of water (250, 350, and 450 mL) in a casserole (Bangqi ceramic, Fuzhou, China), then placed on an induction cooker (Bear, Foshan, China) and cooked for 40, 80, and 120 min, respectively. The specific method was to use the hot pot function key of the induction cooker, adjust the heating power of the induction cooker to 1100 W, cook the sample solution to boiling, and then reduce the power to 300 W, cooking for 40, 80, and 120 min, respectively. Then, the mixtures were refrigerated overnight at −80 °C and dried in a vacuum freeze dryer (Yaxingyike, LGJ-12N, Beijing, China).

### 2.2. Chemicals and Reagents

All standard reference materials, including 3, 4-dihydroxybenzoic acid, 2, 5-dihydroxybenzoic acid, 2, 4-dihydroxybenzoic acid, vanillic acid, 4-nitrophenyl-α-D-glucopyranoside, gallic acid, and trichloroacetic acid were purchased from Energy Chemical in Zesheng City, Anhui Province. Catechin, epicatechin, syringic acid, vanillin, 4-coumaric acid, ferulic acid, iso-ferulic acid, sinapic acid, rosmarinic acid, and kaempferol were purchased from the Aladdin reagent website. Naringin, α-Glucosidase, pancreatic lipase, 4-methylumbelliferyl oleate, orlistat, acarbose, and Folin–Ciocalteu’s phenol reagent were purchased from the Sigma-Aldrich reagent website. Neochlorogenic acid was from purchased Beijing Zhongke Quality Inspection Biotechnology Co., Ltd. Methanol, ascorbic acid, aluminum nitrate, sodium hydroxide, sodium nitrite, salicylic acid, ethanol, 30%H_2_O_2_, FeSO_4_·7H_2_O, hydrochloric acid, sulfuric acid, ammonium molybdate, sodium tripolyphosphate, disodium hydrogen phosphate, sodium dihydrogen phosphate, potassium ferricyanide, ferric chloride, and 1,1-diphenyl-2-trinitrophenylhydrazide were all analytically pure.

### 2.3. Extraction of Polyphenols

#### 2.3.1. Extraction of Soluble-Free and Soluble-Conjugated Phenolic Fractions of Yam

Soluble-free polyphenols (FPs) and soluble-conjugated polyphenols (AHP+BHP) of yam were extracted following a previously reported procedure [19,32,33,34,35] with minor modifications. Yam powder was added to a 70% methanol solution containing 0.1% HCl (*v*/*v*) (solid–liquid ratio 1:10), shaken with a shaker (bluepard, DKA-1C, Shanghai, China) for 2 h at room temperature, and ultrasonicated using an ultrasonic cleaner (Skymen, Shenzhen, China) for 15 min. The mixture was centrifuged at 6000 rpm for 30 min using a centrifuge (CenLee, Changsha, China), and the supernatant was collected. The residue was re-extracted twice to obtain the crude extract. Repeating the above steps twice, the solvent was added to the residue, and the supernatant was collected after using ultrasound for 15 min to obtain the crude extract.

To obtain the FPs, 1.5 mL of crude extract were concentrated using a freezing centrifuge concentrator (Jiaimu, Auto R1-Plus, Beijing, China). Then, 0.5 mL of hydrochloric acid aqueous solution (pH = 2) were added to the concentrated crude extract. The resulting mixture was subjected to extraction three times using 1 mL of ether/ethyl acetate solution (DE/EA, *v*/*v*, 1:1), ultra-sonicated for 15 min, and centrifuged at 3000 rpm for 10 min to collect FP, which retained the remaining aqueous phase.

The aqueous phase was hydrolyzed in 125 μL of 10 M NaOH (final NaOH concentration of 2 M) and stirred at room temperature overnight. The hydrolysate was acidified with 105 μL concentrated hydrochloric acid to pH 2, and the liberated polyphenols were extracted three times with DE/EA, as mentioned above. The collected organic layer was the soluble alkali-hydrolyzable conjugated phenolic fraction (BHP), which retained the remaining aqueous phase.

The remaining aqueous extract was added to 146 μL of concentrated hydrochloric acid (final HCl concentration of 2 M) and heated at 85 °C for 1 h. The conjugated phenolics were extracted three times with DE/EA, as mentioned above. The collected organic layer, a soluble acid-hydrolyzable conjugated phenolic fraction (AHP), was dried using a vacuum freeze dryer.

#### 2.3.2. Extraction of Insoluble Bound Phenolic Fractions of Yam

Following a previously reported procedure [24,32], 1.5 g of residue after crude extraction of soluble phenolics were added to 10 mL of 2 mol/L HCl and heated at 85 °C for 1 h. The pH of the resulting solution was adjusted to 2 after cooling, and the solution was centrifuged at 6000 rpm for 5 min. The supernatant was extracted with 10 mL of DE/EA six times. Then, the collected supernatant was dried using a vacuum freeze dryer to obtain an insoluble acid-hydrolyzable bound phenolic fraction (ABP).

Insoluble alkali-hydrolyzed bound phenols (BBPs) were extracted following previously reported methods [19,32] with slight modifications. First, 1.5 g of residue after crude extraction of soluble phenolics were added to 10 mL of 2 mol/L NaOH and shaken for 4 h. Then, the pH of the mixed solution was adjusted to 2 with hydrochloric acid, and the solution was centrifuged at 6000 rpm for 5 min. The supernatant was extracted with 10 mL of DE/EA six times, as mentioned above. Then, the collected supernatant was dried using a vacuum freeze dryer to obtain BBPs.

### 2.4. HPLC Analysis of Chinese Yams

The composition of polyphenols in raw Ruichang yam was preliminarily investigated by HPLC (Agilent 1260 infinity II, Agilent, USA) with a variable wavelength UV detector (VWD) at 280 nm. Chromatographic separations were conducted at 25 °C on an Agilent C18 column (4.6 mm, 250 mm), with a sample injection of 7 μL. Mobile phase B is a 95% methanol acetonitrile solution containing 5% acetonitrile, and mobile phase D is a 5% formic acid solution. The gradient elution is as follows: 0–40 min, 100–20% D; 40–48 min, 20–0% D; 48–50 min, 0% D; 50–52 min, 0–100% D; 52–55 min, 100% D.

### 2.5. Analysis of Bioactive Components of Chinese Yams

#### 2.5.1. Determination of Total Phenolic Content

The total phenolic content (TPC) was determined following the method reported by Tang Tang [35]. Precisely, 25 μL of sample and gallic acid solution (100–6.25 μg/mL) were transferred into a 96-well plate, and 125 μL of 0.2 mol/L Folin–Ciocalteu phenol reagent were added, mixed, and reacted in the dark for 10 min. Then, 125 μL of 7.5% Na_2_CO_3_ were added to the mixture and reacted in the dark at room temperature for 60 min. The absorbance of the mixture was measured at 765 nm using a microplate reader (Thermo Scientific Multiskan FC, Shanghai, China). The measurements were repeated three times in parallel for all samples. The results were expressed as microgram gallic acid equivalents/g of dried yam powder (μg GAE/DW g).

#### 2.5.2. Determination of the Total Flavonoid Content

The content of total flavonoids (TFC) in polyphenol components was determined using a slightly modified Al^3+^ colorimetric method [36]. Briefly, 25 μL of sample solution or standard solution of catechins (500–61.25 μg/mL) and 110 μL of 0.66 M sodium nitrite solution were mixed in a 96-well plate and allowed to stand for 5 min. Afterward, 15 μL of 0. 375 M Al (NO_3_)_3_ solution were added to the resulting mixture and allowed to stand for 5 min. Then, 100 mL of 0.5 M NaOH solution were added to the mixture and reacted for 10 min. The absorbance of the mixed solution was measured at 510 nm. The measurements were repeated three times in parallel for all samples. The results were expressed as microgram catechin equivalents/g of dried yam powder (μg CAE/DW g).

### 2.6. Evaluation of Antioxidant Activities of Ruichang Yam

#### 2.6.1. Radical Scavenging Activity (DPPH) Assay

The free radical activity of polyphenols was determined using the modified method [36]. Precisely, 50 μL of ascorbic acid methanol solution (190–25 μg/mL) and each sample were added to 200 μL of 140 μg/mL 1,1-diphenyl-2-trinitrophenylhydrazide solution and incubated in a dark place at room temperature for 30 min. The parallel experiment was repeated three times. The absorbance of the obtained solution was measured at 517 nm by a microplate reader. The clearance rate of DPPH was calculated using Equation (1). The results were reported as μg ascorbic acid equivalents/g of dried yam powder (μg AAE/DW g).

(1) $$E=\frac{A_{0}-(A_{2}-A_{1})}{A_{0}},$$

where E is the radical scavenging clearance rate, A_0_ is the absorbance measured by a 50 μL sample solvent in place of a sample solution, A_1_ is the absorbance measured by 1,1-diphenyl-2-trinitrophenylhydrazide solution replaced by 200 μL methanol, and A_2_ is the absorbance of the test sample.

#### 2.6.2. Hydroxyl Radical Activity (OH) Assay

The clearance rate of polyphenol hydroxyl radicals was measured following a previously reported method [37]. First, 50 μL of sample solution were added to 50 μL of 0.03 mol/L FeSO_4_ solution, 50 μL of salicylic acid ethanol solution, and 50 μL of 1% H_2_O_2_ solution. After the sample was mixed, the resulting solution was incubated in a 37 °C incubator for 30 min in the dark. The parallel experiment was repeated three times, and the absorbance of the solution was measured at 510 nm using a microplate reader. The clearance rate of OH was calculated using Equation (2). The results are reported as μg ascorbic acid equivalents/g of dried yam powder (μg AAE/DW g).

(2) $$E=\frac{\left(A_{0}-A_{1})-(A_{2}-A_{3}\right)}{A_{0}-A_{1}},$$

where E is the polyphenol hydroxyl radical clearance rate, A_0_ is the absorbance of 50 μL of sample solvent instead of the blank group of sample solution, A_1_ is the absorbance of the aqueous solution instead of the H_2_O_2_ solution in the blank control group, A_2_ is the absorbance of the test sample, and A_3_ is the absorbance of the sample control group replacing the H_2_O_2_ solution with water.

#### 2.6.3. Total Antioxidant Activity (T-AOC) Assay

The total antioxidant capacity of polyphenols of Chinese yams in each group was determined using a previously reported method [38]. Precisely, 0.4 mL of sample or ascorbic acid solution (2000–125 μM) were mixed with 4 mL of 0.6 M sulfuric acid, 4 mL of 4 mM ammonium molybdate solution, and 4 mL of 28 mM sodium tripolyphosphate. Afterward, the mixture was incubated in a 95 °C water bath for 90 min, and its absorbance was measured at 695 nm using a UV-visible spectrophotometer (PERSEE, TU-1810PC, Beijing, China) at room temperature. The measurements were repeated three times in parallel for all samples. The result was expressed as the number of micromoles of ascorbic acid equivalents per gram of yam (μmoL AAE/DW g).

#### 2.6.4. Total Reducing Activity (T-RC) Assay

The total reducing capacity of polyphenols in each group of yams was measured following a previously reported procedure [39]. First, 1 mL of ascorbic acid (1000–62.5 μM) or sample solution, 2.5 mL of sodium phosphate-buffered solution (0.2 mM pH = 6.6), and 2.5 mL of 1% potassium ferricyanide solution (*w*/*v*) were transferred to a centrifuge tube and heated in a water bath at 50 °C for 30 min. Then, 2.5 mL of 10% trichloroacetic acid (*w*/*v*) were added at room temperature. The mixture system was centrifuged at 3000 rpm for 10 min, and the supernatant was collected. Then, 2.5 mL of the supernatant were mixed with 2.5 mL of water and 0.5 mL of 0.1% ferric chloride (*w*/*v*). After the mixture was reacted for 10 min, its absorbance was determined at 700 nm using a UV-visible spectrophotometer. The measurements were repeated three times in parallel for all samples. The total reducing activity was expressed as the number of micromoles of ascorbic acid equivalents (AAE) per gram of yam (μmoL AAE/DW g).

### 2.7. Enzyme Inhibition

#### 2.7.1. α-Glucosidase Inhibition Assay

In the assay of α-Glucosidase activity of samples in vitro using a microplate reader [40], acarbose was selected as the positive control of the sample. The sample was dissolved in a sodium phosphate buffer (pH 6.8) to form a sample solution with the required concentration gradient (0–6 mg/mL). Then, 50 μL of sample solution with different concentrations were collected and mixed with 50 μL of 0.2 U/mL α-Glucosidase solution in 96-well plates. After the mixture was incubated at 37 °C for 10 min, 15 μL of 5 mM 4-nitrophenyl-α-D-glucopyranoside solution were added. Then, the resulting solution was incubated at 37 °C for 10 min, and 100 μL of 0.2 M Na_2_CO_3_ solution were added to terminate the reaction. The absorbance of the solution was immediately measured at 405 nm using a microplate reader. The measurements were repeated three times in parallel for all samples. The inhibition rate was calculated according to Equation (3). The half inhibitory activity of α-Glucosidase was expressed by IC_50_, which is the substrate concentration required for 50% enzyme activity (mg/mL).

(3) $$E=\frac{A_{0}-A_{2}}{A_{0}-A_{1}},$$

where E is the inhibition rate of α-Glucosidase, and A_0_ is the absorbance measured by sodium phosphate-buffered solution in place of the sample solution. A_1_ is the absorbance measured by the α-Glucosidase solution replaced by sodium phosphate-buffered solution in the control group. A_2_ is the absorbance of the test sample.

#### 2.7.2. Pancreatic Lipase Inhibition Assay

The pancreatic lipase inhibitory activity of polyphenols was determined using a fluorescent microplate reader (Thermoscientific, Shanghai, China) [24,41]. Orlistat was selected as the positive of the sample. The sample was dissolved in Tris-HCl (pH 8.0). Then, 25 μL of the sample solution with different concentrations, 25 μL of 0.1 mg/mL pancreatic lipase solution, and 50 μL of 1 μM 4-methylumbelliferyl oleate solution were mixed in a 96-well plate and then incubated at 37 °C for 25 min. The absorbance of the mixture was immediately measured using a fluorescence microplate reader at Ex = 320 and Em = 450 nm. The measurements were repeated three times in parallel for all samples. The inhibition rate was calculated using Equation (4). The inhibitory activity of pancreatic lipase was expressed in IC_50_, as similarly defined for α-Glucosidase.

(4) $$E=1-\frac{A_{2}-A_{3}}{A_{0}-A_{1}},$$

where E is the inhibition rate of pancreatic lipase, and A_0_ is the absorbance measured by the Tris-HCl solution in place of the sample solution in the blank group. A_1_ is the absorbance measured by the Tris-HCl solution replaced by the pancreatic lipase solution in the control group. A_2_ is the absorbance of the test sample, and A_3_ is the absorbance measured by water in place of the Tris-HCl solution in the blank control group.

### 2.8. Data Analysis

Data are expressed as mean ± standard deviation for triplicate experiments. All statistical analyses were conducted using IBM SPSS Statistics 26, and statistical significance was determined using a one-way analysis of variance (*p* < 0.05) with Tukey’s test. The enzyme inhibition rate (IC_50_) was determined using GraphPad Prism 9.3.0. The degree of association between the variables was assessed using Pearson’s correlation coefficient. All the figures were drawn using Origin 2021.

## 3. Results and Analysis

### 3.1. Chemical Composition of Polyphenols in Chinese Yams

**Figure 1:**
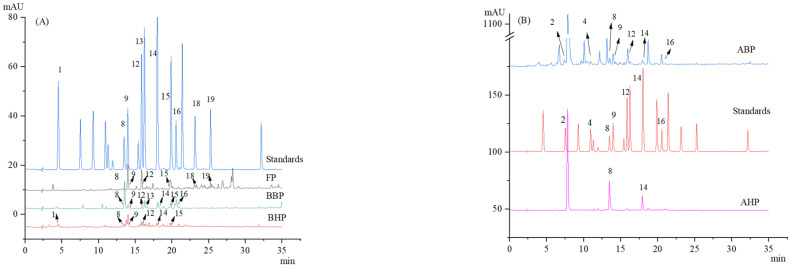
Phenolic compounds in Chinese Ruichang yam. (**A**) The phenolic compounds in FPs, BHPs, and BBPs; (**B**) the phenolic compounds in AHPs and ABPs. FPs: soluble-free polyphenols, BHPs: alkali-hydrolyzed soluble-conjugated polyphenols, AHPs: acid-hydrolyzed soluble-conjugated polyphenols, ABPs: acid-hydrolyzed insoluble-bound polyphenols, and BBPs: alkali-hydrolyzed, bound, conjugated polyphenols. Numbers: 1: gallic acid, 2: 3, 4-dihydroxybenzoic acid, 4: p-hydroxybenzoic acid, 8: 2, 4-dihydroxybenzoic acid, 9: vanillic acid, 12: syringic acid, 13: vanillin, 14: 4-coumaric acid, 15: ferulic acid, 16: sinapic acid, 18: naringin, 19: rosmarinic acid.

The chemical compositions of FPs, BHPs, AHPs, ABPs, and BBPs were investigated. In total, 6, 5,2, 7 and 7 compounds were preliminary detected in FPs, BHPs, AHPs, ABPs, and BBPs by HPLC, respectively (Figure 1). 2, 4-dihydroxybenzoic acid (8), vanillic acid (9), and syringic acid (12) were preliminarily confirmed to be contained in FPs, BHPs, ABPs, and BBPs by comparison of standard products. Additionally, FPs also contained ferulic acid (15), naringin (18), and rosmarinic acid (19). BHPs and BBPs also contained 4-coumaric acid (14) and ferulic acid (15), and vanillin (13) and sinapic acid (16) were found in BBPs. ABPs also contained 4-coumaric acid (14), sinapic acid (16), 3, 4-dihydroxybenzoic acid (2), and p-hydroxybenzoic acid (4). There were relatively few components in AHP, which were 2, 4-dihydroxybenzoic acid (8) and 4-coumaric acid (14) by comparison of standard products.

### 3.2. Effect of Cooking Time on Polyphenols and Flavonoids in Chinese Yams

Phenolics and flavonoids are the largest groups of phenolics in vegetables and are related to antioxidant potential because they act as reducing agents and scavengers of free radicals [42]. Therefore, investigating the effect of cooking time on the stability of bioactive compounds is crucial due to the biological functions of plants. Considering all fraction compounds (FP+BHP+AHP+ABP+BBP), the TPC and TFC of uncooked Ruichang yam were 275.85 ± 8.15 and 112.92 ± 12.09 μg CAE/g, respectively, which were lower than those of cooked samples: 508.62 ± 15.45 μg GAE/g and 144.35 ± 37.65 μg CAE/g, respectively, at 40 min. At 80 min, the TPC and TFC of cooked Ruichang yam were 425.42 ± 24.7 μg GAE/g and 147.06 ± 8.92 μg CAE/g, respectively, which were higher than those of samples cooked for 120 min: 153.04 ± 6.08 μg GAE/g and 83.12 ± 5.92 μg CAE/g dry weight, respectively (Table 1 and Figure S1 and Figure 1A). These results show that cooking times increase the TPC and TFC in the yam. Kido found that heat treatment increased the total phenol content in sweet potato tubers [16]. The possible reason is that heat treatment can soften and break cell components, thus facilitating the release of phenolic compounds [43]. The analysis of uncooked Chinese yams revealed that cooking times of 40, 80, and 120 min had different effects on the TPC and TFC (*p* < 0.05) (Table 1). The variation was possibly due to the variance of phenolic compounds. The factors influencing the contents of polyphenols and flavonoids in samples were the cooking technique, heat transfer medium, and heat transfer intensity [18]. Cooking times of 40 and 80 had a higher TPC and TFC, respectively, compared to cooking for 120 min. The possible reason is that the heat generated by cooking can result in changes in the structure of phenolic compounds [44]. A longer cooking time may lead to the conversion of flavonoids and phenols into other substances.

**Figure 2:**
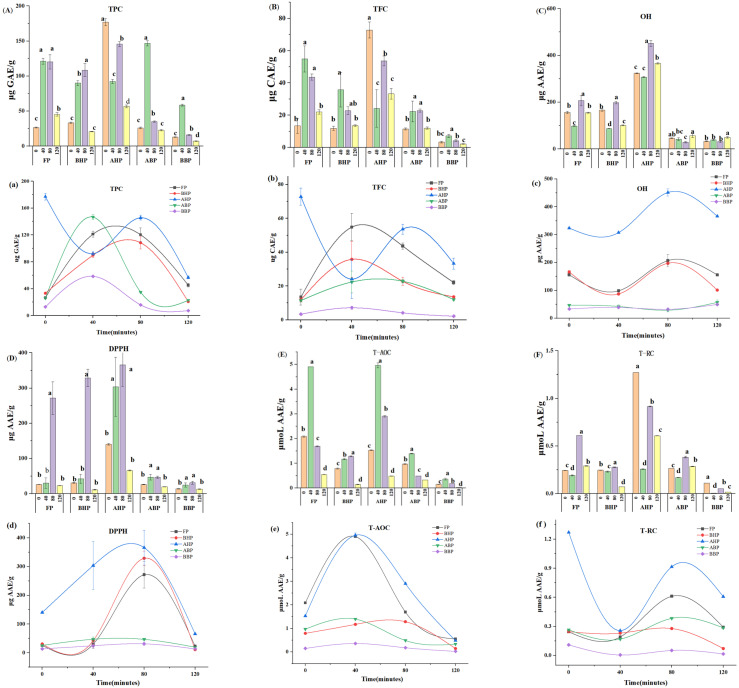
(**a**–**f**) TPC: total polyphenol content, TFC: total flavonoid content, OH: hydroxyl radical activity, DPPH: radical scavenging activity, T-AOC: total antioxidant activity, and T-RC: total reducing activity. For individual bar graphs, (**A**–**F**) represent the values of the TPC, TFC, OH, DPPH, T-AOC, and T-RC of Ruichang yam during cooking time. FP: soluble-free polyphenols, BHPs: alkali-hydrolyzed soluble-conjugated polyphenols, AHPs: acid-hydrolyzed soluble-conjugated polyphenols, ABPs: acid-hydrolyzed insoluble-bound polyphenols, and BBPs: alkali-hydrolyzed, bound, conjugated polyphenols; 0: raw yam, 40: cooked for 40 min, 80: cooked for 80 min, and 120: cooked for 120 min. The data are presented as mean values ± standard deviation (SD) (*n* = 3). According to Tukey’s multiple range test (*p* < 0.05), the mean values indicated with letters (a, b, c, and d) indicate significant differences (*p* < 0.05) between uncooked (0) samples and cooked samples for 40, 80, and 120 min.

Among the three types of polyphenols, including FPs, BHPs+AHPs, and insoluble bound polyphenols (ABPs+BBPs), FPs were significantly affected by cooking times of 40, 80, and 120 min, yielding 23.8, 28.3, and 29.6% of the TPC and 38.0, 29.6, and 26.5% of the TFC (Table 1), respectively. In the samples extracted at three different cooking times, the TPC of FPs gradually decreased with increasing cooking times (Figure 2a,A). When the cooking time was 40 min or 80 min, the TPC of FPs was higher (121.18 ± 4.24, 120.34 ± 10.18 μg GAE/g, respectively, *p* < 0.05). In addition, the TPC of FPs in a sample cooked for 80 min increased by approximately 350% compared with an uncooked sample (26.60 ± 0.65 μg GAE/g, *p* < 0.05). The TPC of FPs in the sample cooked for 120 min (45.37 ± 2.45 μg GAE/g, *p* < 0.05) was the lowest, and it increased by approximately 70% compared with that of an uncooked sample. The changing trend in the TFC in FPs was consistent with that of the TPC (Figure 2b,B). When the cooking time was 40 min, the TFC of FPs (54.86 ± 8.06 μg CAE/g, *p* < 0.05) was the highest (Table 1, Figure S1B), and the TFC of FPs in the sample cooked for 40 min increased by approximately 300% compared to the uncooked sample. The TFC of FPs in the sample cooked for 120 min (22.05 ± 1.35 μg CAE/g, *p* < 0.05) was the lowest (Table 1, Figure S1B), but it increased by about 64% compared with that of the uncooked yam. Some similar studies have shown that the TPC of purple potato was positively correlated with the heat treatment time within 15–25 min, and the TPC decreased with the increase in time after the boiling time exceeded 25 min [45]. After steaming 12 types of potatoes for 30, 40, and 50 min, the researchers found that 11 kinds of potatoes had the highest TPC and six kinds of potatoes had the highest TFC after steaming for 40 min, while the TPC and TFC were relatively low after steaming for 30 and 50 min [46].

As shown in Table 1, the TPCs and TFC of BHPs+AHPs were relatively high during cooking (about 35.8–59.7% and 41.5–56.4%, respectively). The TPCs (254.09 ± 12.88 μg GAE/g) and TFC (76.42 ± 5.47 μg CAE/g) of BHPs+AHPs were the highest when the boiling time was 80 min (Figure S1C). Furthermore, when cooked for 80 min, the TPC in BHPs+AHPs was significantly higher than that of the uncooked sample (210.35 ± 6.12 μg CAE/g), and the TFC (84.72 ± 6.33 μg CAE/g) of the cooked sample was significantly lower than that of the uncooked samples (Figure S1C). Compared with the TPC and TFC of BHPs+AHPs in uncooked yams, the TPC of BHPs+AHPs after cooking for 80 min increased by 20%, and the TFC decreased by 9.7% (Figure S1C). The TPC and TFC in the AHPs of uncooked yams were higher than those in the AHPs of cooked yams. However, the TPC in the BHPs of the yam cooked for 80 min was significantly higher than that of the uncooked yam, increasing by 69%, while the TFC was reduced by 26%. Therefore, the TPC and TFC of BHPs+AHPs were higher when the cooking time was 80 min. Studies have shown that the acid hydrolysis process at high temperatures leads to the loss of some phenolic substances and the formation of furan derivatives [35]. When concentrations exceed 3000 mg/kg, these compounds reacted with Folin–Ciocalteu reagents [47], resulting in higher polyphenol content than the actual value. Our experimental result showed that the TPC (177.06 ± 5.13 μg GAE/g) of AHPs in uncooked yam was the highest, indicating that the concentration of furan derivatives produced during acid hydrolysis was below 3000 mg/kg. The TPC of BHPs and AHPs after cooking had similar changes during the cooking process. When the cooking time was 80 min, BHPs and AHPs exhibited the highest TPC, which were 108.47 ± 9.32 and 145.63 ± 3.55 μg/g, respectively. However, when the cooking time was 40 min, BHPs exhibited the highest TFC (35.8 ± 10.66 μg CAE/g), accounting for 24.8% of the TFC of all cooked yam-type polyphenols. AHPs in the samples cooked for 80 min exhibited the highest TFC (53.62 ± 3.02 μg CAE/g), accounting for 36.46% of the TFC of all yam-type polyphenols (Table 1). These data showed that the TPC of AHPs and BHPs were the highest when boiled for 80 min, while the TFC of BHPs was the highest when boiled for 40 min.

In the cooked yam (Table 1), the TPC and TFC in ABPs+BBPs were lower than those in FPs and BHPs+AHPs, accounting for 12.0–40.3% and 17.1–20.5%, respectively. When the cooking time was 40 min, the ABPs+BBPs exhibited the highest TPC (205.21 ± 4.93 μg GAE/g) and TFC (29.54 ± 7.34 μg CAE/g) (*p* < 0.05). The TPC (30.16 ± 1.35 μg GAE/g) and TFC (14.20 ± 0.84 μg CAE/g) of ABPs+BBPs in the sample cooked for 120 min were the lowest (Figure S1D). The TPC in ABPs and BBPs also had similar changes during the cooking process (Figure 2a,b,A,B). When the cooking time was 40 min, the TPC in ABPs and BBPs were the highest (*p* < 0.05), which were 146.67 ± 3.66 and 58.54 ± 1.27 μg GAE/g, accounting for 28.8% and 11.5% of the TPC of all yam-type polyphenols, respectively. In addition, when the cooking time was 80 min, the TFC in ABPs (22.84 ± 1.02 μg CAE/g) was the highest, accounting for 15.5% of the TFC of all yam-type polyphenols. The TFC of BBPs in the yam sample cooked for 40 min (7.15 ± 0.94 μg CAE/g) was the highest, accounting for 5.0% of TFC of all yam-type polyphenols.

### 3.3. Effect of Cooking Time on the Antioxidant Activity of Chinese Yam Polyphenols

Radical scavenging activities play important roles in reducing the risk of chronic diseases [48]. Due to the different reaction mechanisms, four assays were conducted to evaluate the radical scavenging properties of a selected sample. Relative OH, DPPH, T-AOC, and T-RC were examined in this study. The analysis results of the cooked Chinese yam are presented in Table 1. All fraction compounds were considered, including FPs, BHPs, AHPs, ABPs, and BBPs. When the cooking time was 80 min, the DPPH (1044.47 ± 139.38 μg AAE/ g), OH (916.81 ± 48.28 μg AAE/g), T-AOC (12.78 ± 0.18 μg AAE/g), and T-RC (2.24 ± 0.02 μmoL AAE/g) had the highest values (Figure S1A). The DPPH, OH, T-AOC, and T-RC values increased by 77, 20, 56, and 4% compared to the uncooked yam samples. The DPPH, OH, T-AOC, and T-RC scavenging activities of the cooked samples were enhanced. The T-AOC value of all fraction compounds peaked at a cooking time of 40 min, while the remaining three parameters peaked at a cooking time of 80 min.

FPs, BHPs+AHPs, and ABPs+BBPs are presented in Table 1. Among them, the main component of free radical scavenging activity in the yam sample was soluble conjugated polyphenols. In the DPPH, OH, T-AOC, and T-RC assays, the scavenging activities of soluble conjugated phenolics accounted for 57.9–77.3%, 64.1–70.8%, 41.2–64.1%, and 53.1–71.0% of the respective total scavenging activities. Generally, soluble-bound phenolics contain BHPs and AHPs. As shown in Figure 2c–f,C–F, in five type phenolics, FPs, BHPs, AHPs, ABPs, and BBPs, the values of the DPPH, OH, T-AOC, and T-RC of AHP were the highest, indicating that AHPs have strong free radical scavenging activity; however, ABPs and BBPs exhibited low values, indicating that their free radical scavenging activity is small. The TPC and TFC in AHPs were higher (Figure 2a,b,A,B), possibly because alkali hydrolysis can only break the ester bond between phenols and the matrix, while acid hydrolysis can break both the ester bond and glycoside bond [49]. Therefore, the TPC and TFC of AHPs are higher than those of the other four types of yam polyphenols.

The analysis of cooked Chinese yam (Table 1) revealed that FPs and BHPs+AHPs of the yam cooked for 80 min had the highest DPPH (271.57 ± 46.31 and 694.87 ± 85.51 μg AAE/g, respectively), OH (206.79 ± 21.81 and 394.60 ± 2.67 μg AAE/g, respectively), and T-RC (0.61 ± 0.00 and 0.44 ± 0.01 μmoL AAE/g, respectively) values (Figure S1B). The values of DPPH, OH, and T-RC scavenging activities of FPs in the sample cooked for 80 min were 10.0, 1.3, and 2.5 times that of uncooked samples, respectively. Compared with uncooked yam, the T-RC of the soluble conjugated phenolics in the yam cooked for 80 min was reduced by 20%, but the DPPH and OH were 4.0 and 1.3 times that of the uncooked yam sample, respectively. The OH values of FPs, BHPs, and AHPs first increased and then decreased with increasing cooking time (Figure 2c,C). When the cooking time was 80 min, the OH values of FPs, BHPs, and AHPs reached their maximum, measuring 206.79 ± 21.81, 197.55 ± 5.31, and 451.42 ± 12.73 μg AAE/g (*p* < 0.05) (Figure S1B), and were higher than for uncooked yam (OH:156.05 ± 5.53, BHP:166.16 ± 5.31, and AHP: 323.67 ± 1.08 μg AAE/g, *p* < 0.05). However, the value of OH of ABPs and BBPs initially decreased and then increased with the extension of time. When the cooking time was 120 min, the OH values of ABPs and BBPs were the highest (57.02 ± 8.39 and 49.55 ± 3.75 μg AAE/g, respectively), which were slightly higher than those of uncooked samples (46.53 ± 1.50 and 33.18 ± 1.49 μg AAE/g, respectively, *p* < 0.05) (Figure 2c,C). All the DPPH of the five types of phenolics in the cooked sample initially increased and then decreased with the increase in cooking time (Figure 2d,D). The DPPH value of each phenolic in the sample cooked for 80 min was the highest. In the yam samples cooked for 80 min, the T-RC values of five types of phenolics were the highest (Figure 2f,F). These data showed that the five phenolic substances boiled for 80 min had higher DPPH and TRC activities, which may also be related to the high total phenol content of the polyphenols boiled for 80 min.

The T-AOC was evaluated in the yam sample (Table 1). Among soluble FPs, BHPs+AHPs, and ABPs+BBPs, FPs accounted for 25.8–38.3% of the T-AOC of all yam-type polyphenols. AHPs+BHPs played an important role in the T-AOC scavenging activity, accounting for 41.2–64.1% of the T-AOC of all yam-type polyphenols. Insoluble-bound phenolics accounted for 10.1–22.9% of the T-AOC. The T-AOC values of FPs, BHPs+AHPs, and ABPs+BBPs were the highest when the cooking time was 40 min, measuring 4.90 ± 0.00, 6.13 ± 0.14, and 1.75 ± 0.05 μmoL AAE/g (*p* < 0.05), and these values were approximately 2.3, 2.6, and 1.5 times those of the uncooked yam samples, respectively (Table 1, Figure S1). When the cooking time was 120 min, the T-AOC values of the three polyphenols were the lowest (0.55 ± 0.00, 0.63 ± 0.01, and 0.35 ± 0.00 μmoL AAE/g, respectively). To sum up, the T-AOC values of FPs, BHPs, AHPs, ABPs, and BBPs for a cooking time of 120 min were the lowest, and except for BHPs, the T-AOC values of the other four phenolics for a cooking time of 40 min were the highest (Figure 2e,E).

### 3.4. In Vitro α-Glucosidase and Pancreatic Lipase Activity

**Figure 3:**
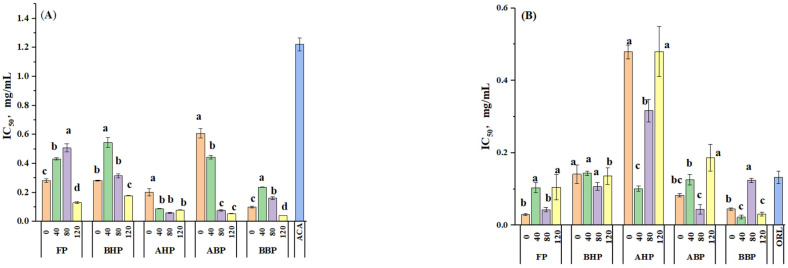
Half-inhibitory concentration (IC_50_, mg/mL) for α-Glucosidase (**A**) and pancreatic lipase inhibitory activity (**B**). FPs: soluble-free polyphenols; BHPs: alkali-hydrolyzed soluble-conjugated polyphenols; AHPs: acid-hydrolyzed soluble-conjugated polyphenols; ABPs: acid-hydrolyzed insoluble-bound polyphenols; BBPs: alkali-hydrolyzed, bound, conjugated polyphenols. ACA: acarbose; ORL: orlistat; 0: uncooked Chinese Ruichang yam; 40: cooked for 40 min; 80: cooked for 80 min; 120: cooked for 120 min. Different letters in the same group indicate significant differences (mean ± standard deviation, n = 3, *p* < 0.05).

Acarbose was used as a positive control to evaluate the inhibitory activity of phenols on α-Glucosidase (Figure 3A). Acarbose is a drug that has been used to treat type 2 diabetes for approximately 30 years [50]. The half-inhibitory concentration of α-Glucosidase in all groups was lower than that of positive control acarbose (IC_50_ = 1.223 ± 0.045 mg/mL), indicating that yam polyphenols had outstanding α-Glucosidase inhibitory activity. In traditional Chinese medicine, yams are usually added to a prescription to treat diabetes [51]. Small molecular constituents in yams have been confirmed to have α-Glucosidase inhibitory activity, such as batatasin I and trans-N-p-coumaroyl tyramine [2]. Among them, BBPs, ABPs, and AHPs of yams cooked for 120 min exhibited the strongest α-Glucosidase inhibitory activity (IC_50_ = 0.041 ± 0.001, 0.055 ± 0.002, 0.079 ± 0.001 mg/mL), while ABPs of uncooked yams exhibited the lowest inhibitory activity (IC_50_ = 0.608 ± 0.033 mg/mL). Because the content and compositions in the polyphenols of yams are different, they show different α-Glucosidase inhibitory activity. When the cooking time was 40 min or 80 min, the IC_50_ of FPs, BHPs, and BBPs of the cooked yam significantly increased compared to the uncooked yam, while the IC_50_ of AHPs and ABPs decreased significantly. Additionally, the IC_50_ of FPs, BHPs, AHPs, ABPs, and BBPs significantly decreased when the boiling time was 120 min. The longer the cooking time, the better the α-Glucosidase inhibitory activity. The reason may be that heat treatment makes the yam produce other molecules through a thermalization reaction, which has better-inhibiting α-Glucosidase activity. The structural change and enzyme activity relationship of yam polyphenols under heating conditions will be the focus of subsequent research. Our results showed that cooking affected the α-Glucosidase inhibitory activity of yam polyphenols. The cooking effect was more significant when the boiling time was 120 min, and the hypoglycemic function was also enhanced.

Pancreatic lipase is a key enzyme in digesting triacylglycerol, which can hydrolyze 50–70% of the total dietary fat in the digestive system and convert triglycerides into glycerol monoesters and free fatty acids [52]. Pancreatic lipase is an enzyme that is involved in fat digestion. Natural pancreatic lipase inhibitors play an important role in the treatment of obesity [25]. Orlistat is an anti-obesity medication approved by the US Food and Drug Administration (FDA) [53]. The 4-methylumbelliferyl oleate substrate was used to evaluate the inhibitory activity of the polyphenol extract on pancreatic lipase (Figure 3B). The half-inhibitory concentration of more than half of the species of polyphenols was lower than that of orlistat (IC50 = 0.132 ± 0.0172 mg/mL), indicating that some polyphenol extracts had excellent inhibitory activity on pancreatic lipase. Compared with the uncooked sample, the IC_50_ of pancreatic lipase of AHPs and BBPs with the samples cooked for 40 min decreased, the IC50 of pancreatic lipase of BHPs, AHPs, and ABPs with the samples cooked for 80 min decreased, and the IC50 of pancreatic lipase of BBPs with the samples cooked for 120 min decreased (Figure 3B). Heat treatment can soften and break cell components, thus facilitating the release of phenolic compounds [43]. The yams released different polyphenols at different cooking times; thus, the pancreatic lipase inhibition was different. As the cooking time increased, the IC_50_ of pancreatic lipase of FPs, BHPs, and ABPs first decreased and then increased. The inhibitory activity of pancreatic lipase first increased and then decreased, while the IC50 pancreatic lipase of BBPs was on the contrary, and the IC_50_ of pancreatic lipase of AHPs successively increased. Too long or too short a cooking time could affect the enzyme activity of polyphenols. Too short a cooking time may result in incomplete softening of yam cells, while too long a cooking time may completely soften cells, converting phenolic substances into other phenolic compounds, leading to differences in polyphenol composition among different groups. It is also possible that specific phenolic acids have strong pancreatic lipase inhibitory activity, such as Coumarins and ferulic acid, and have the inhibitory activity of pancreatic lipase [54]. The amount of them directly affects enzyme activity. To sum up, BBPs of yams cooked for 40 (0.022 ± 0.005 mg/mL) and 120 (0.03 ± 0.005 mg/mL) min exhibited the strongest pancreatic lipase inhibitory activity. Secondly, FPs (0.044 ± 0.013 mg/mL) and ABPs (0.043 ± 0.006) of yams cooked for 80 min had higher pancreatic lipase inhibitory activity, and their lipid-lowering activity was the best.

In this study, the low concentration of bound phenols released by acid or alkaline hydrolysis of the yam (ABPs and BBPs) can inhibit α-Glucosidase and pancreatic lipase. This may be related to the composition of phenolic compounds. However, there are fewer bound phenols in yam polyphenols, and ABPs and BBPs contain sinapic acid, which was not detected in free and conjugated polyphenols. However, even if these phenols can inhibit these two enzymes in vitro, they may not be able to achieve the concentration of inhibition in vivo. However, existing studies have shown that appropriate cooking methods, enzymatic hydrolysis, or fermentation can increase the release of bound phenols. For example, the treatment of Miang extract with tannase facilitates the formation of gallate catechin ester and gallate and has a potential inhibitory effect on pancreatic lipase [55]. Microwave cooking increases the TPC in the bound phenolic extracts in lentils [56]. Fermentation facilitates the release of oligomers and bioactive compounds (protocatechuic acid, gallic acid, and catechol) [57]. Colonic fermentation can enable the release of bound polyphenols from wheat bran dietary fiber [58], thereby increasing the bioavailability of polyphenols. The bound polyphenols in carrot insoluble dietary fiber can be released through mixed solid fermentation, which is higher than that released through alkaline hydrolysis [59].

### 3.5. Pearson Correlation Analysis

**Figure 4:**
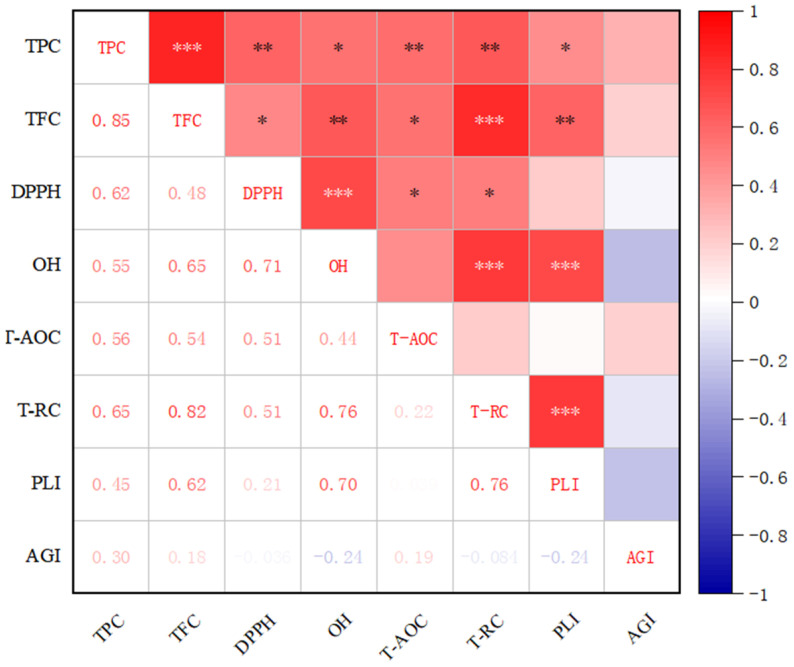
Pearson’s correlation coefficient chart. The darker red, the stronger the positive correlation, and the darker blue, the stronger the negative correlation. TPC: total polyphenol content, TFC: total flavonoid content, OH: hydroxyl radical activity, DPPH: radical scavenging activity, T-AOC: total antioxidant activity, and T-RC: total reducing activity. AGI: half-inhibitory concentration for α-Glucosidase; PLI: half-inhibitory concentration of pancreatic lipase. “*” is the correlation between the two test indicators in the results of correlation coefficient measurement (*p* ≤ 0.05), and “**” is the significant correlation between the two test indicators in the results of correlation coefficient measurement (*p* ≤ 0.01). “***” is the very significant correlation between the two test indicators in the results of correlation coefficient measurement (*p* ≤ 0.001).

Pearson’s correlation coefficient was used to evaluate the correlation among polyphenol indexes (Figure 4). The TPC was positively correlated with the TFC (R = 0.85, *p* ≤ 0.001), DPPH (R = 0.62, *p* ≤ 0.01), OH (R = 0.55, *p* ≤ 0.05), and TRC (R = 0.65, *p* ≤ 0.01). The content of total flavonoids was also positively correlated with the index of oxidation activity. The OH was positively correlated with the DPPH (R = 0.71, *p* ≤ 0.001) and T-RC (R = 0.76, *p* ≤ 0.001). The PLI was positively correlated with the OH (R = 0.70, *p* ≤ 0.001), TFC (R = 0.62, *p* ≤ 0.01), and TRC (R = 0.76, *p* ≤ 0.001). However, the AGI was negatively correlated with the PLI and OH, and the correlation was insignificant. In summary, flavonoid and polyphenol contents and antioxidant activity indexes were strongly correlated, and pancreatic lipase inhibition was strongly correlated with the TFC, OH, and T-RC.

## 4. Discussion

Polyphenols are key active components in plants known for their health-promoting effects. The TPC is used to estimate the content of phenolic compounds in a sample. Phenolic compounds exhibit redox properties, which are mainly responsible for their antioxidant properties. In this study, free phenolic, conjugated phenolic, and bound phenolic compounds were the phenolic compounds determined in raw and cooked yams. Studies have shown that the content of total phenolic compounds in different varieties of yam varies from 1.65 to 10.168 mg/g (DW) [18,60,61]. The total soluble phenolic content measured in this study was 0.236 mg GAE/g, which was lower than that in previous reports (Table 1). These differences may be attributed to yam varieties, growing environments, harvesting and storage times, and extraction and determination methods. Only a few studies have reported on the effect of cooking treatment on the phenolic compound composition of yams. Gong [18] reported that the phenolic compound composition of yams after processing via atmospheric steaming (1.32 ± 0.13 mg GAE/g dry weight), atmospheric cooking (1.17 ± 0.04 mg GAE/g dry weight), high-pressure steaming (1.28 ± 0.15 mg GAE/g dry weight), high-pressure cooking (1.08 ± 0.05 mg GAE/g dry weight), and microwaving (1.22 ± 0.03 mg GAE/g dry weight) was lower than that of raw yams (1.65 ± 0.06 mg GAE/g dry weight). However, in this study, we found that soluble phenolic compounds and flavonoids significantly increased during cooking (Table 1). In this study, Ruichang yam polyphenols were extracted from a mixture of solids and liquids after cooking yams, which was different from the previously reported method of cooking yams. However, during the cooking process, the heat could soften and break cell components, facilitating the release of phenolic compounds [43]. Therefore, part of the soluble polyphenols released during the boiling process of the yam remain in the liquid, resulting in an increase in the TPC and TFC after the yam is boiled.

To the best of our knowledge, this study is the first to determine the polyphenol content, whether free phenolic, conjugated phenolic, or insoluble bound phenolic, in Ruichang yam. We observed different correlations between the polyphenol content and various antioxidant activities. In general, the presence of polyphenols and flavonoids enhanced the bioactive characteristics of yams related to their antioxidant capacity. Acid or alkali treatment broke the chemical bond between phenolic compounds and the substrate [49], facilitating the release of polyphenols. The furan derivatives and their derivatives produced through acid hydrolysis exhibited a certain degree of antioxidant activity in DPPH experiments [62]. The interaction between plant polyphenols and the pectin and cellulose molecules of plant cell walls has a complex effect on the bioactivity of polyphenols [63,64], which may play a synergistic or antagonistic role. In addition, the antioxidant activity of polyphenols is also related to their composition. Chlorogenic acid, caffeic acid, ferulic acid, and other polyphenols are important components of the antioxidant activity of potatoes [65]. Studies have shown that vanillic acid and kaempferol have good DPPH radical scavenging activity [66].

In addition to antioxidant activity, phenolic extracts can inhibit enzyme inhibition mechanisms in the body. Studies have shown that Chinese purple yam polyphenols significantly inhibit α-Glucosidase [67]. The phenolic components of Korean yam (Dioscorea opposita) can effectively inhibit pancreatic lipase in vitro [68]. To confirm polyphenol activity in biological models, we treated alpha-glucosidase and pancreatic lipase using free phenols and phenols released from conjugated and bound forms. The phenolic compounds tested in both raw and cooked Chinese yams exhibited good activity in inhibiting α-Glucosidase. In addition, the longer the cooking time (120 min) (Figure 4), the better their inhibitory effect. Although the polyphenol content was the lowest when the cooking time was 120 min, the phenolic acid released at this same time might have strong α-Glucosidase inhibitory activity. Some phenolic components, such as rutin, chlorogenic acid, epicatechin, ferulic acid, and quercetin, have good α-Glucosidase inhibitory activity [67]. The Pearson correlation analysis revealed that pancreatic lipase had a stronger positive correlation with the TFC than the TPC. It may be that some phenolic and flavonoid compounds of yam polyphenols have a strong inhibitory activity on pancreatic lipase. For example, flavonoids exhibited stronger inhibitory pancreatic lipase activity than flavanols and isoflavones [69]. Abu [70] screened 15 common phenolic compounds and found that only gallate, gallatechin, and gallatechin gallate had high lipase inhibition potential. Furthermore, (+) catechin, (−) epicatechin, (+) gallocatechin, and (−) epigallocatechin had pancreatic lipase-inhibited activity. The flavonoid compounds released by different cooking treatments varied, resulting in different inhibitory pancreatic lipase activities. This variation indicates that the two enzymes of free, conjugated, and insoluble-bound phenols in yams exhibit inhibitory effects, possibly due to their significant antioxidant activity or a mixture of polyphenols produced through chemical hydrolysis and boiling treatment. As a result, further research is needed to confirm these processes.

## 5. Conclusions

In this study, cooking for 40 and 80 min enhanced the TPC and TFC of FPs, BHPs, ABPs, and BBPs of yams, and the TPC and TFC had a positive correlation with the OH, DPPH, T-RC, and T-AOC. Eleven compounds were detected in yam polyphenol extracts by the HPLC: syringic acid existed in FPs and ABPs, vanillic acid existed in FPs, BHPs, and ABPs, 2, 4-dihydroxybenzoic acid existed in FPs, AHPs, and BBPs, 4-coumaric acid existed in AHPs and BBPs, and sinapic acid existed in ABPs and BBPs. Additionally, the TPC and TFC of soluble-conjugated phenolics were the main phenolic substances in yam polyphenols. All of the polyphenols had good α-Glucosidase inhibitory activity, and only some polyphenol components had good pancreatic lipase inhibitory activity. Too long or too short a cooking time could affect the enzyme activity of polyphenols. As the cooking time increased, the α-Glucosidase inhibitory activity of all types of polyphenols also increased, and the ABPs and BBPs had the strongest α-Glucosidase inhibitory when cooking for 120 min. BBPs showed the best inhibitory activity of pancreatic lipase when the cooking time was 40 or 120 min. The IC_50_ of pancreatic lipase was positively correlated with the OH, TFC, and TRC. These data suggest that we can design the cooking time of yams to maximize their activity. In domestic cooking, a shorter cooking time (40 to 80 min) can obtain a higher TPC and antioxidant activity. Appropriately increasing the cooking time (120 min) can better prevent type II diabetes. Therefore, preliminary studies have shown that yam foods with different effects can be cooked by controlling the heating time, making them suitable for consumption by different populations.

## Figures and Tables

**Table 1:** Effects of uncooked and cooked (40, 80, and 120 min) states on indexes (TPC, TFC, DPPH, OH, T-AOC, and T-RC) of Chinese Ruichang yam.

		TPC (μg GAE/g)	TFC (μg CAE/g)	Radical Scavenging Activity (μg AAE/g)		Radical Scavenging Activity (μmoL AAE/g)	
				DPPH	OH	T-AOC	T-RC
0	FPs	26.60 ± 0.65 ^a^	13.43 ± 4.65 ^a^	26.01 ± 0.38	156.05 ± 5.53 ^a^	2.08 ± 0.03 ^a^	0.24 ± 0.00 ^a^
	BHPs	33.29 ± 0.99 ^a^	11.87 ± 1.30 ^a^	30.66 ± 1.11	166.16 ± 5.31 ^a^	0.79 ± 0.03 ^a^	0.25 ± 0.00 ^a^
	AHPs	177.06 ± 5.13 ^a^	72.85 ± 5.02 ^a^	140.53 ± 2.42 ^a^	323.67 ± 1.08	1.53 ± 0.01 ^a^	1.27 ± 0.00 ^a^
	BHPs+AHPs	210.35 ± 6.12	84.72 ± 6.33	171.2 ± 3.53	489.84 ± 6.38	2.32 ± 0.04	1.52 ± 0.00
	ABPs	25.94 ± 1.15 ^a^	11.43 ± 0.59 ^a^	25.84 ± 0.47 ^a^	46.53 ± 1.50	0.97 ± 0.01 ^a^	0.27 ± 0.00 ^a^
	BBPs	12.96 ± 0.26 ^a^	3.33 ± 0.52 ^a^	13.95 ± 0.56 ^a^	33.18 ± 1.49	0.15 ± 0.01 ^a^	0.11 ± 0.00 ^a^
	ABPs+BBPs	38.90 ± 1.41	14.77 ± 1.11	39.79 ± 1.03	79.71 ± 3.00	1.12 ± 0.02	0.38 ± 0.00
	Total	275.85 ± 8.18	112.92 ± 12.09	236.99 ± 4.94	725.60 ± 14.91	5.52 ± 0.08	2.14 ± 0.00
40	FPs	121.18 ± 4.24 *	54.86 ± 8.06 *	30.06 ± 14.77	98.44 ± 1.30 *	4.90 ± 0.00 *	0.19 ± 0.01 *
	BHPs	89.92 ± 3.49 *	35.8 ± 10.66 *	42.21 ± 12.69	87.22 ± 0.18 *	1.17 ± 0.03 *	0.23 ± 0.01 *
	AHPs	92.32 ± 2.78 *	24.14 ± 11.58 *	303.77 ± 83.6 *	307.38 ± 2.49	4.96 ± 0.10 *	0.26 ± 0.00 *
	BHPs+AHPs	182.24 ± 6.27	59.95 ± 22.24	345.98 ± 96.29	394.60 ± 2.67	6.13 ± 0.14	0.49 ± 0.01
	ABPs	146.67 ± 3.66 *	22.39 ± 6.39 *	47.16 ± 8.08 *	42.30 ± 6.10	1.39 ± 0.01 *	0.17 ± 0.00 *
	BBPs	58.54 ± 1.27 *	7.15 ± 0.94 *	24.54 ± 6.36 *	38.73 ± 2.80	0.36 ± 0.04 *	0.01 ± 0.00 *
	ABPs+BBPs	205.21 ± 4.93	29.54 ± 7.34	71.71 ± 14.43	81.04 ± 8.90	1.75 ± 0.05	0.17 ± 0.00
	Total	508.62 ± 15.45	144.35 ± 37.65	447.75 ± 125.48	574.07 ± 12.87	12.78 ± 0.18	0.85 ± 0.02
80	FPs	120.34 ± 10.18 *	43.66 ± 1.85 *	271.57 ± 46.31 *^,a^	206.79 ± 21.81 *^,a^	1.69 ± 0.02 *^,a^	0.61 ± 0.00 *^,a^
	BHPs	108.47 ± 9.32 *^,a^	22.80 ± 2.44	328.98 ± 24.69 *^,a^	197.55 ± 5.31 *^,a^	1.28 ± 0.02 *^,a^	0.28 ± 0.00 *^,a^
	AHPs	145.63 ± 3.55 *^,a^	53.62 ± 3.02 *^,a^	365.89 ± 60.82 *	451.42 ± 12.73 *^,a^	2.90 ± 0.03 *^,a^	0.92 ± 0.00 *^,a^
	BHPs+AHPs	254.09 ± 12.88	76.42 ± 5.47	694.87 ± 85.51	648.97 ± 18.04	4.19 ± 0.05	1.19 ± 0.01
	ABPs	35.01 ± 0.99 *^,a^	22.84 ± 1.02 *	46.83 ± 2.96 *	28.87 ± 3.66 *	0.49 ± 0.00 *^,a^	0.38 ± 0.01 *^,a^
	BBPs	15.98 ± 0.64 *^,a^	4.14 ± 0.58 ^a^	31.20 ± 4.59 *	32.19 ± 4.76	0.18 ± 0.00 ^a^	0.05 ± 0.00 *^,a^
	ABPs+BBPs	50.99 ± 1.63	26.98 ± 1.60	78.02 ± 7.55	61.06 ± 8.43	0.66 ± 0.00	0.44 ± 0.01
	Total	425.42 ± 24.7	147.06 ± 8.92	1044.47 ± 139.38	916.81 ± 48.28	6.54 ± 0.07	2.24 ± 0.02
120	FPs	45.37 ± 2.45 *^,a^	22.05 ± 1.35 ^a^	22.95 ± 0.34	155.50 ± 2.05 ^a^	0.55 ± 0.00 *^,a^	0.29 ± 0.00 *^,a^
	BHPs	20.73 ± 0.23 ^a^	13.58 ± 0.51 ^a^	11.19 ± 0.91	101.06 ± 2.19 *^,a^	0.14 ± 0.01 *^,a^	0.07 ± 0.00 *^,a^
	AHPs	56.78 ± 2.04 *^,a^	33.29 ± 3.23 *	65.78 ± 1.40 ^a^	366.18 ± 3.14 *^,a^	0.48 ± 0.01 *^,a^	0.61 ± 0.00 *^,a^
	BHPs+AHPs	77.50 ± 2.27	46.86 ± 3.74	76.98 ± 2.30	467.24 ± 5.33	0.63 ± 0.01	0.68 ± 0.00
	ABPs	22.80 ± 1.02 ^a^	12.02 ± 0.66 ^a^	19.57 ± 0.36 ^a^	57.02 ± 8.39 ^a^	0.33 ± 0.00 *^,a^	0.29 ± 0.00 *^,a^
	BBPs	7.36 ± 0.33 *^,a^	2.18 ± 0.17 ^a^	13.36 ± 0.67 ^a^	49.55 ± 3.75 *^,a^	0.02 ± 0.00 *^,a^	0.02 ± 0.00 *^,a^
	ABPs+BBPs	30.16 ± 1.35	14.20 ± 0.84	32.93 ± 1.04	106.57 ± 12.15	0.35 ± 0.00	0.3 ± 0.00
	Total	153.04 ± 6.08	83.12 ± 5.92	132.86 ± 3.68	729.32 ± 19.52	1.53 ± 0.02	1.27 ± 0.01

0: uncooked Chinese Ruichang yam; 40: cooked for 40 min; 80: cooked for 80 min; 120: cooked for 120 min. FPs: soluble-free polyphenols; BHPs: alkali-hydrolyzed soluble-conjugated polyphenols; AHPs: acid-hydrolyzed soluble-conjugated polyphenols; ABPs: acid-hydrolyzed insoluble-bound polyphenols; BBPs: alkali-hydrolyzed, bound, conjugated polyphenols. AHPs+BHPs: soluble-conjugated; ABPs+BBPs: insoluble-bound. TPC: total polyphenol content, TFC: total flavonoid content, OH: hydroxyl radical activity, DPPH: radical scavenging activity, T-AOC: total antioxidant activity, and T-RC: total reducing activity. The values (average ± standard deviation) followed by “*”, “^a^”, respectively, indicate a significant difference compared with uncooked yam and yam cooked for 40 min, according to Tukey’s multiple range test (*p* < 0.05).

## Data Availability

The original contributions presented in the study are included in the article/Supplementary Material, further inquiries can be directed to the corresponding author.

## Supplementary Materials

The following supporting information can be downloaded at: https://www.mdpi.com/article/10.3390/foods14010014/s1, Figure S1: The effect of cooking time on free polyphenols, conjugated polyphenols, and bound polyphenols; Table S1: Effect of cooking time on the half-inhibitory concentration (IC50, mg/ml) for α-Glucosidase and pancreatic lipase inhibitory activity.
