# Supplementary material for: The Impact of Cooking on Antioxidant and Enzyme Activities in Ruichang Yam Polyphenols
Source: Foods. 2024 Dec 25;14(1):14. doi: 10.3390/foods14010014 (PMC11719820; doi:10.3390/foods14010014)
Supplement: Supplementary file 1 [file foods-14-00014-s001.zip › foods-3348954-supplementary.pdf]

Table S 1. Effect of cooking time on the half-inhibitory concentration (IC50, mg/ml) for α-Glucosidase and pancreatic lipase inhibitory activity.

Type		Half-inhibitory concentration of α-Glucosidase (IC_{50} mg/mL)	Half-inhibitory concentration of pancreatic lipase (IC_{50} mg/mL)
FP	0	0.281±0.012*	0.029±0.003*
	40	0.432±0.008*	0.104±0.014
	80	0.507±0.028*	0.043±0.006*
	120	0.13±0.007*	0.105±0.035
BHP	0	0.282±0.002*	0.141±0.026
	40	0.545±0.034*	0.144±0.006
	80	0.315±0.014*	0.107±0.011
	120	0.179±0.003*	0.136±0.023
AHP	0	0.201±0.025*	0.479±0.019*
	40	0.088±0.002*	0.101±0.007
	80	0.059±0.002*	0.317±0.031*
	120	0.079±0.001*	0.479±0.069*
ABP	0	0.608±0.033*	0.082±0.005
	40	0.444±0.013*	0.126±0.015
	80	0.077±0.004*	0.044±0.013*
	120	0.055±0.002*	0.186±0.037
BBP	0	0.1±0.005*	0.045±0.003*
	40	0.237±0.001*	0.022±0.005*
	80	0.16±0.009*	0.124±0.006
	120	0.041±0.001*	0.03±0.005*
Acarbose		1.223±0.045	______________
Orlistat		______________	0.132±0.017

FPs: soluble free polyphenols; BHPs: alkali-hydrolyzed soluble conjugated polyphenols; AHPs: acid-hydrolyzed soluble conjugated polyphenols; ABPs: acid-hydrolyzed insoluble bound polyphenols; BBPs: alkali-hydrolyzed bound conjugated polyphenols. 0: uncooked Chinese Ruichang yam; 40: cooked for 40 min; 80: cooked for 80 min; 120: cooked for 120 min. The dates is average ± standard deviation, n = 3), “ * ” indicates significant difference compared with the positive control ($P < 0.05$).

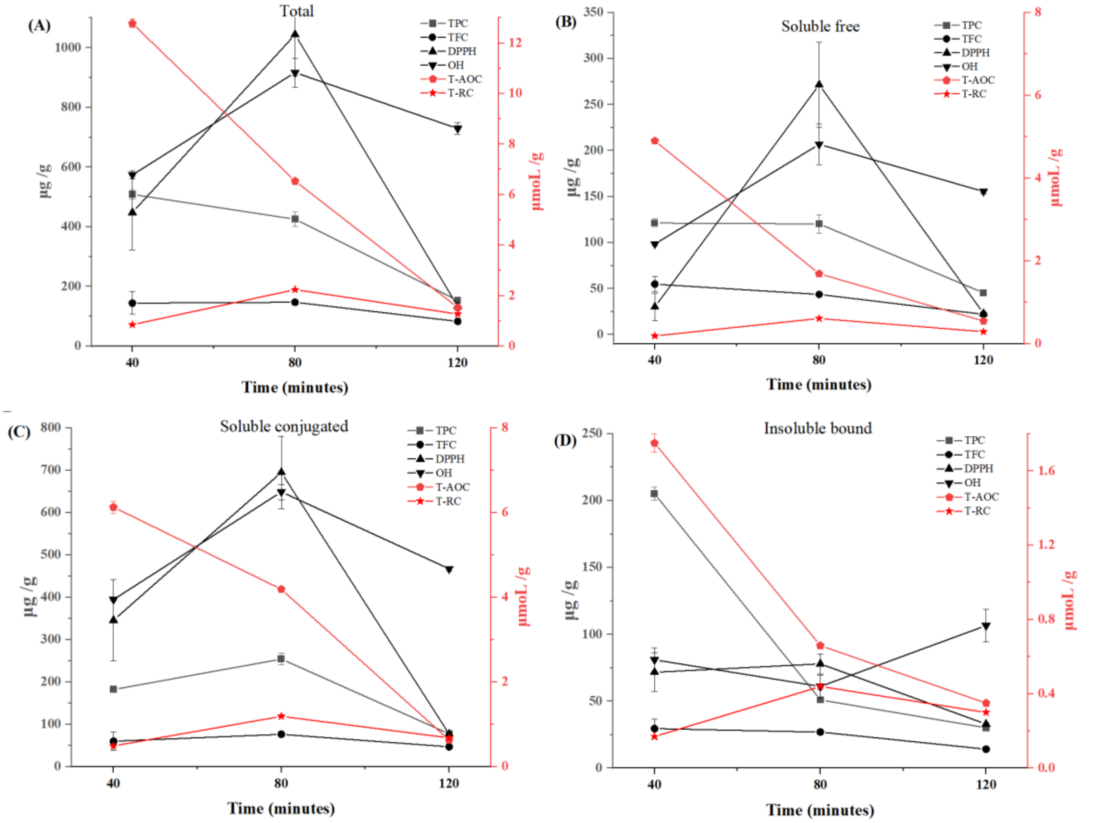

Figure S1. The effect of cooking time on free polyphenols, conjugated polyphenols, and bound polyphenols. A–D, all fraction polyphenols (FPs+BHPs+AHPs+ABPs+BBPs), soluble free polyphenols (FPs), soluble conjugated polyphenols (BHPs+ABPs), and insoluble bound polyphenols (ABPs+BBPs) of cooked Ruichang yam. The data are average values ± standard deviation (SD), $n = 3$. TPC: total polyphenol content, TFC: total flavonoid content, OH: hydroxyl radical activity, DPPH: radical scavenging activity, T-AOC: total antioxidant activity, and T-RC: total reducing activity. FPs: soluble free polyphenols; BHPs: alkali-hydrolyzed soluble conjugated polyphenols; AHPs: acid-hydrolyzed soluble conjugated polyphenols; ABPs: acid-hydrolyzed insoluble bound polyphenols; BBPs: alkali-hydrolyzed bound conjugated polyphenols.
